# Enhancement of Live Food Nutritional Status with Essential Nutrients for Improving Aquatic Animal Health: A Review

**DOI:** 10.3390/ani10122457

**Published:** 2020-12-21

**Authors:** Nur Amalina Samat, Fatimah Md Yusoff, Nadiah W. Rasdi, Murni Karim

**Affiliations:** 1 Laboratory of Aquatic Animal Health and Therapeutics, Institute of Biosciences, Universiti Putra Malaysia (UPM), Serdang 43400, Selangor, Malaysia; amalinasamat@yahoo.com; 2 Laboratory of Sustainable Aquaculture, International Institute of Aquaculture and Aquatic Sciences, Port Dickson 71050, Negeri Sembilan, Malaysia; fatimamy@upm.edu.my; 3 Department of Aquaculture, Faculty of Agriculture, Universiti Putra Malaysia (UPM), Serdang 43400, Selangor, Malaysia; 4 School of Fisheries and Aquaculture Sciences, Universiti Malaysia Terengganu, Kuala Terengganu 21300, Terengganu, Malaysia; nadiah.rasdi@umt.edu.my; 5 Institute of Tropical Biodiversity and Sustainable Development, Universiti Malaysia Terengganu, Kuala Terengganu 21300, Terengganu, Malaysia

**Keywords:** live food, enrichment, nutrients, nutritional quality, aquatic health

## Abstract

**Simple Summary:**

A highly nutritious quality diet that is readily accepted and digested is essential for better growth and development of aquaculture species. Most newly hatched fish and shrimp depend on live food as an important basic diet. Copepods are considered the nutritional benchmark diet for a wide range of marine fish larvae. However, *Artemia* and rotifers are often favored as starter feed, despite their inferior nutritional values in comparison to copepods. Therefore, *Artemia*, rotifers, and other live foods are commonly enriched with nutrients such as fatty acids, vitamins, minerals, and probiotics to imitate the copepod’s level of essential nutrients to improve rearing success for fish and crustacean larvae.

**Abstract:**

At the present time, no artificial larval diet is capable of entirely fulfilling the dietary requirements of several larval fish and crustacean species. Zooplankton live food is the basic foundation of fish larviculture, and successful rearing of fish larvae still heavily depends on an adequate supply of nutritious live food. Despite being important, the production protocols of copepods and cladocerans (*Moina*) are still underdeveloped in hatcheries. Rotifers and *Artemia* are the most commonly used live foods. However, these live foods are evidently lacking in crucial nutrient constituents. Hence, through nutrient enrichment, live food with the nutritional profile that meets the requirements of fish larvae can be produced. With the aim to maximize the effectiveness of production to optimize profitability, it is important to evaluate and improve culture techniques for the delivery of micro- and macro-nutrients as feed supplements to larvae in aquaculture systems. Bioencapsulation and enrichment are the evolving techniques in aquaculture that are commonly employed to enhance the nutritional quality of live food by integrating nutrients into them, which subsequently improves the growth, survival, and disease resistance of the consuming hosts. This review aims to highlight some of the approaches and methods used to improve the nutritional quality of live food by modifying their nutrient composition, which could have immense promise in the enhancement of aquatic animal health.

## 1. Introduction

Larvae of many aquatic species either have complete dependence on zooplankton live food as a basal diet, or they have significantly better performance when started on live food [1]. Live food is commonly regarded as “living capsules of nutrition”, rich in proteins, vitamins, carbohydrates, minerals, amino acids, and fatty acids [2]. As a superior nutritional prey, some zooplankton contain high levels of digestive enzymes [3] and are capable of producing appetite-stimulating effects on larvae [4]. Live food organisms are able to swim freely in the water column, thereby being constantly accessible to finfish and crustacean larvae [5,6]. Their jerking movements are likely to stimulate larval feeding responses [7]. On the contrary, formulated feeds often accumulate on the water surface or some slowly sink to the bottom, whereby becoming less accessible to larvae [5]. Zooplankton such as rotifers and *Artemia* are by far the most commonly utilized live food in the cultivation of finfish and crustaceans [8].

Substitution of live food by formulated diets has been emphasized [9]. However, the sole application of a formulated diet may seem like a far-fetched idea due to low its digestibility and the deterioration of water quality [6,7]. Even though the use of live food in larval rearing has been reported to improve larval growth performance, survival, and disease resistance [1,10,11], the cultivation and management of live food for aquatic production is costly and unpredictable [12]. Multiple studies have demonstrated the success of total live food replacement or reduction in aquaculture [9,13]. It is important to understand the nutritional requirements of fish larvae in order to facilitate the optimization of diets and feeding protocols, which may subsequently enhance larval quality [7,14]. Consequently, several studies have emphasized developing practical methods to improve the nutritional status of live food with essential nutrients [15,16,17,18,19,20].

By taking advantage of primitive feeding characteristics, the manipulation of the nutritional status of zooplankton is achievable by pre-feeding them through the so-called “bioencapsulation” or “enrichment” protocols. Through enrichment techniques, essential nutrients lacking in zooplankton, prophylactics, and therapeutics can be delivered to fish larvae via zooplankton live food. The application of enriched live food is reflected in enhanced growth, survival, stress tolerance, and microbial diversity for a variety of aquatic species [19,21,22,23,24]. A very important aspect of live food enrichment is its reproducibility and predictability, which are crucial in commercial hatcheries. Hence, it is necessary to constantly produce high-quality live food on a large scale [15]. However, producing enriched live food with consistent levels of the important nutrients can be complex. This review aimed to emphasize the significance of live food and the implementation of different enrichment techniques to incorporate nutrients such as minerals, vitamins, microalgae, lipids, and probiotics to enhance the nutritional status of the live food and to subsequently boost the health of the aquatic animals.

## 2. Enrichment with Fatty Acids

Highly unsaturated fatty acids (HUFA) with 20 or more carbon atoms are one of the major sources of metabolic energy during the embryonic and pre-feeding larval stages in fish. However, these energy sources rapidly declined during the endogenous feeding stage [25]. The n-3 series HUFA docosahexaenoic acid (DHA, 22:6n-3) and eicosapentaenoic acid (EPA, 20:5n-3), and the n-6 series HUFA arachidonic acid (ARA, 20:4n-6), play significant roles in fish larval development; thus, the deficiency of HUFA may impair fish growth, reproduction, and survival, causing pale or swollen liver, myocarditis, intestinal steatosis, lordosis, fin erosion, and shock syndrome [26]. HUFA are synthesized in very small concentrations from their precursors alpha-linolenic acid (ALA, 18:3n-3) and linoleic acid (LA, 18:2n-6) [27] due to the lack of delta-5 and delta-6 desaturases and elongases in marine fish larvae [28]. Therefore, HUFA must be incorporated through live foods such as copepods, rotifers and *Artemia* to meet the requirements for larval growth [27]. The requirements of HUFA in fish and crustaceans have been widely studied. The effects of dietary HUFA in the juveniles of golden pompano (*Trachinotus ovatus*) [29], yellowtail (*Seriola dumerili*) [30], Asian seabass (*Lates calcarifer*) [31], and Pacific white shrimp (*Litopenaeus vannamei*) [32] are among the most recently published studies.

Enrichment of live food with commercial oil emulsion (Super Selco, DHA Selco, Selco S.presso) is a common practice [33,34,35,36,37,38,39,40]. Several studies have assessed the dietary fatty acid profiles of copepods and enriched *Artemia* [39,41,42]. The predominant fatty acids in copepods are DHA, EPA, and palmitic acid, while DHA, EPA, and oleic acid are the predominant fatty acids in *Artemia* enriched with Super Selco and DHA Selco [41]. Apart from the absolute amount of HUFA, the dietary DHA/EPA ratio is suggested to impact the normal growth and development of certain fish species [43,44]. The average DHA/EPA ratio for copepods ranged between 1.83 and 5.5 whereas the DHA/EPA ratio for *Artemia* enriched with DHA Selco ranged from 1.4 to 2.2 [41,42]. The DHA/EPA ratio of *Artemia* enriched with Super Selco at 600 mg/L for 16 h was reported at 0.2 [39], whilst enrichment at 200 and 300 mg/L for 24 and 20 h, respectively, brought about 0.68 and 0.3 DHA/EPA ratio, respectively [38,41]. Altogether, *Artemia* enriched with DHA Selco recorded a higher DHA/EPA ratio than that of Super Selco. The instability of HUFA and the catabolism of these compounds by *Artemia* in addition to low DHA retention efficiency in *Artemia* during the first 24 h post enrichment might be the contributing factors to this variation [38,41,45]. Commercial emulsions are more stable and effective as the primary emulsions are mainly made from HUFA-rich fish oils and emulsified with egg yolk and seawater. However, these forms of enrichment formula are low in efficiency but are cheap alternatives in developing countries [7]. Higher DHA and EPA contents and DHA/EPA ratio were recorded in the freshwater cladoceran *Moina micrura* enriched with commercial emulsion (Maxepa MERCK, Delhi, India) in addition to gelatine, egg yolk, and Celin [19]. Modifications of dietary fatty acid compositions of rotifers and *Artemia* should be made in line with those of copepods.

Boosting of the nutritional status of rotifers [46,47], *Artemia* [48,49], copepods [50,51,52,53,54,55,56,57], and *Moina* [58] through algal enrichment techniques is a common practice to boost the quality of the otherwise nutrient-deficient feed. Microalgae is a rich source of HUFA and polyunsaturated fatty acids (PUFA) [43,59,60]. It is easier to control the essential fatty acid (EFA) composition of enrichment emulsions when microalgae-derived oil is used in comparison with purified fish oils [61]. Due to the high cost and difficulty in producing, concentrating, and storing live microalgae, the development of different forms of microalgae as a replacement to live microalgae has become a major focus of research [62]. A cheaper microalga paste has been used in aquaculture practice as an alternative to live microalgae [63]. Rotifers fed on microalgal pastes (*Nannochloropsis oculata* and *Chlorella vulgaris*) at equal quantities were rich in palmitic acid, linoleic acid, and EPA after 48 h of exposure to the microalgal diet. However, the DHA content was only recorded at 6 mg/g dry weight (DW). Nevertheless, the DHA content was enough to improve the growth, development, and stress resistance of fish larvae [64]. This study underlined the importance of enriching rotifers fed to larvae with multiple microalgal species over monospecific diets.

A previous study investigated the fatty acid composition of rotifers enriched with a mixture of DHA-enriched *C. vulgaris* (Super fresh Chlorella V12, SV, Chlorella Industry, Tokyo, Japan) and DHA emulsion (Bio Chromis, Chlorella Industry, Tokyo, Japan) for 12 h [43]. The DHA content in enriched rotifers increased from 0.1 to 15.4% and the DHA/EPA ratio was highest in the treatment. DHA was found to be dominant in rotifers enriched with DHA- and arachidonic acid (AA)-rich oils extracted from the dinoflagellate *Crythecodinium* sp. and the fungus *Mortierella alpine*, respectively, in addition to EPA-rich marine oil [65]. Rotifers have a better retention rate of EPA compared to DHA, regardless of the ratio in their enrichment [44]. Enrichment of rotifers can be achieved either through short-term enrichment (alteration of the lipid content of the rotifers just before larval feeding) and long-term enrichment (feeding of rotifers on a complete diet) [66,67]. Enriched DHA was stable in rotifers at 10 °C for at least 24 h post-enrichment under starving conditions, whereas a higher temperature of 20 °C significantly decreased the DHA level during starvation [68]. Rotifers emptied their gut at a reduced rate as culture temperature decreased from 26 °C to 4 °C [69]. Moreover, microalgae are often added to the enrichment formula to promote “green water” to maintain the nutritional quality of zooplankton [64,70,71]. The larvae of rainbow trout (*Oncorhynchus mykiss*) [72], Russian sturgeon (*Acipenser gueldenstaedtii*) [73], Atlantic sturgeon (*Acipenser oxyrinchus*) [74], caspian kutum (*Rutilus frisii kutum*) [75], yellowtail flounder (*Limanda ferruginea*) [65], gilthead seabream (*Sparus aurata*) [28], and greater amberjack (*S. dumerili*) [24], whitefish (*R. kutum*) fry [76], and juvenile milkfish (*Chanos chanos*) [77] have been reared with live food enriched with essential fatty acids.

The high contents of EPA, DHA, and some digestive enzymes in copepods are among the important properties that make them a superior live food to *Artemia* and rotifers [6]. Therefore, it is recommended to enrich zooplankton in order to meet copepod HUFA levels. The enrichment emulsions are commonly prepared using commercial emulsions such as DHA Selco and Super Selco. To meet the copepod DHA/EPA ratio, it is recommended that *Artemia* and rotifers be enriched with DHA Selco. Even though studies on HUFA enrichment in *Moina* are fairly limited, a study has successfully enriched *Moina* with Maxepa. Additionally, HUFA enrichment can be performed using microalgae, either live or pastes. It is recommended that microalgae pastes be used as a cheaper alternative to live microalgae, and the application of multiple microalgal species over monospecific diets would be very beneficial. Moreover, a combination of commercial emulsions and microalgae in an enrichment mixture would be advantageous in terms of enhancing the DHA/EPA ratio.

## 3. Enrichment with Vitamins

### 3.1. Vitamin C

Vitamin C (VC) plays a vital role in the growth, immune response [78], hematology and histology [79], antioxidant and enzyme activities [80], reproduction [81], wound healing [82], and response to stressors [83] of fish and crustaceans. The addition of VC in aquaculture practices has been proven to enhance the growth performance, antioxidant defense system, and production of many aquatic animals including freshwater prawn (*Macrobrachium malcolmsonii*) [80] and kuruma shrimp (*Marsupenaeus japonicus* Bate) [84]. Enrichment of *Artemia* with of ascorbyl-6-palmitate for 24 h was observed to significantly reduce the mortality rate of seabream larvae [85]. However, it is necessary to note that a high dose of vitamin supplementation may cause lipid peroxidation in fish tissues under oxidative stress conditions [86]. The dietary requirement of VC may decrease with increased size [87]. Lack of VC can lead to structural deformities and internal hemorrhaging [88]. Some aquatic animals including the majority of crustacean and fish species are unable to synthesize VC because of the absence of the enzyme L-gluconolactone oxidase, which is essential for the last step of VC biosynthesis [89]. Hence, they depend on feed for a constant supply of VC.

Brown and Hohmann [90] reported a significant effect of the algal growth phase on the percentage of ascorbic acid in the culture of *Isochrysis* sp. However, the results need to be contextualized with the standard hatchery practice for algal production, the balance of other nutrients in the microalga, and the dietary requirements of the aquatic animal [90]. The enrichment of *A. franciscana* with the microalgae *Isochrysis galbana* for 72 h had a favorable impact on the amount of ascorbic acid in *Artemia* depending on the ascorbic acid content of *I. galbana* [45]. Moreover, the enrichment of rotifers with ascorbyl palmitate improved the assimilation of ascorbic acid when the percentage of ascorbyl palmitate incorporation in the enrichment media increased, thus suggesting that the ascorbic acid levels in *Artemia* nauplii can be manipulated via bioencapsulation of different ascorbyl palmitate concentrations [91]. The positive effects of feeding VC-enriched live food were reported on the larvae of milkfish (*C. chanos*) [92] and climbing perch (*Anabas testudineus*) [19], Senegalese sole (*Solea senegalensis*) [93], and Patagonian red octopus paralarvae (*Enteroctopus megalocyathus*) [94]. Generally, boosting of ascorbic acid content in zooplankton live food through the algal enrichment technique at a commercial hatchery yielded a lower ascorbic acid concentration than in the laboratory, probably due to differences in the culture conditions [91]. Different species and enrichment procedures resulted in different ascorbic acid levels in zooplankton [6], but the enrichment of microalgae with VC had been reported to increase the concentration of VC in *Artemia* [93], rotifers [95], and copepods [96]. However, it is crucial to have prior knowledge on the natural content of vitamins in microalgae before they are subjected to any enrichment procedure. There may be variations due to species and culture conditions with regards to light and nutrient conditions; protocols for harvesting, processing, and storage; extraction; and analysis [97]. The compositional data of vitamin contents in microalgae need to be compared to the dietary needs of the consuming aquatic animals. Unfortunately, the dietary requirements for larval or juvenile animals that feed on zooplankton are poorly understood [97]. Moreover, the concentrations of vitamins in the intermediary zooplankton and the transfer efficiency in the food chain are the area that requires further research [97,98].

The pre-enrichment of microalgae with VC would be beneficial to a large group of filter-feeding zooplankton that utilize microalgae as a major source of food. Therefore, enrichment of zooplankton such as *Artemia*, rotifers, and copepods with microalgae to boost their VC levels is a practical approach. The levels of VC in zooplankton can be manipulated by enriching them with ascorbyl palmitate at different concentrations. However, when utilizing microalgae in an enrichment procedure, several factors including culture conditions, harvesting, processing, and storage protocols, as well as extraction and analysis must be taken into account, as these may greatly impact the VC levels in zooplankton live food.

### 3.2. Vitamin A

Vitamin A (VA) is a vital nutrient for fish as the compound cannot be synthesized de novo. Many VA or retinoid forms are available as dietary supplements including retinol (the alcohol form of VA), retinal (the aldehyde form), retinoic acid (the acid form), and retinyl acetate and retinyl palmitate (the ester form) [99]. VA hypervitaminosis can cause skeletal malformations in different vertebral regions, as well as cephalic malformations in jaw and fin complexes in other marine fishes, including the larvae of gilthead sea bream (*S. aurata*) [100], red sea bream (*Chrysophrys major*) [101], European sea bass (*Dicentrarchus labrax*) [102], Japanese flounder (*Paralichthys olivaceus*) [103,104], striped trumpeter (*Latris lineata*) and post larvae [99], and summer flounder (*Paralichthys dendatus*) [105]. The positive effect of dietary VA were reported on the juveniles of Nile tilapia (*Oreochromis niloticus*) [106] and spotted grouper (*Epinephelus coioides*) [107], on-growing gibel carp (*Carassius auratus gibelio*) [108], rainbow trout (*O. mykiss*) fry [109], and on Japanese flounder (*P. olivaceus*) larvae [110].

It was reported that VA deficiency in *Artemia* caused incomplete migration of the eye during Atlantic halibut (*Hippoglossus hippoglossus* L.) larvae metamorphosis [111]. Another study reported a higher concentration of VA in Atlantic halibut when fed with marine copepod than those fed with *Artemia* [112], probably because *Artemia* contains carotenoids in the form of cryptoxanthin or canthaxanthin, while the source of carotenoids in copepods is lutein and astaxanthin [61]. Thus, it is appropriate to enrich *Artemia* with copepod-type carotenoids. Furthermore, it was reported that the vitamin content in rotifers was below the levels found in copepods, but generally still within the range required by fish larvae [113]. Considering the limited amount of literature on fish larval nutrient requirements, the nutrient levels of copepod are considered as a target for the enrichment [114]. Moreover, VA was not detected in rotifers fed with basic diet containing Baker’s yeast, suggesting a need for further enrichment with pure VA [113]. It is reported that the total VA accumulation in rotifers was independent to the dose [17,114], but dependent to the added doses in the case of *Artemia* [115]. However, another study demonstrated otherwise [100]. Studies have demonstrated the possibility of zooplankton enrichment using liposomes [115] and commercial emulsion [100,116], in which retinyl palmitate was the dominant form of the retinoids in the emulsions and in the enriched zooplankton. However, Monroig et al. [115] reported a noteworthy result of the poor efficiency of the commercial emulsion in *Artemia*, despite it containing retinyl palmitate, probably due to a partial degradation during the enrichment process influenced by different abiotic conditions. Moreover, the emulsion quality and properties; differences in the strains and batches of the zooplankton used; or the stages of development, metabolic capability, and filtration rates may contribute to the enrichment efficiency of the emulsions [17]. Therefore, the application of vesicles such as liposomes to bioencapsulate VA in live food is a promising approach. Liposomes as a retinyl palmitate carrier to zooplankton provide extra protection for VA from oxidation, and thus a higher amount of retinyl palmitate can be bioencapsulated in the zooplankton to be fed to aquatic animals [115].

Given the limited number of studies on fish larval nutrient requirements, the VA levels of copepods are considered as a target for enrichment. *Artemia* can be enriched with VA in the form of lutein and astaxanthin to meet the copepod VA levels. Even though the vitamin content in rotifers was below the levels found in copepods, it is still within the range required by fish larvae. Several studies reported that zooplankton such as *Artemia* can be enriched with liposomes and commercial emulsion. However, the partial degradation of VA during the enrichment process must be taken into account when employing commercial emulsions for zooplankton enrichment. Various studies on the applications of vitamin C and A in live food have been compiled in Table 1.

## 4. Enrichment with Minerals

### 4.1. Selenium

Selenium (Se) is a vital trace element for many aquatic animals; however, the line between requirement and toxicity is obscured [121]. Se acts as an antioxidant [122] and plays an important role in the regulation of the thyroid hormone metabolism and the endocrine system [123], cell signaling, growth, and survival [124]. Since Se cannot be produced naturally by living organisms, it has to be obtained from the diet [125]. Supplementation of Se to the basal diet is reported to enhance the growth of grouper (*Epinephelus malabaricus*) [126], Atlantic cod (*G. morhua*) larvae [127], and crucian carp (*C. gibelio*) [128]. It also plays a significant role in the detoxification of cadmium and green synthesized silver nanoparticle (Ag-NP) toxicity in abalone (*Haliotis discus hannai*) and Nile tilapia (*O. niloticus*), respectively [129,130].

Enrichment of live food with selenium has been performed on rotifers [127] and *Artemia* [15] to meet the copepod Se levels. This element is transferred in the food chain to zooplankton and fish larvae [6]. It is reported that the concentration of Se in rotifers can be over 30 times lower than in copepods [113,127], which does not meet the mineral requirements of fish [131]. Therefore, it is necessary to establish an enrichment method to mass-produce rotifers with adequate amounts of the micronutrient. Ponce et al. [125] employed different rotifer enrichment strategies by using three forms of Se: selenite, selemethionine, and selenized yeast. The uptake of selenite increased linearly with exposure time while selemethionine was the dominant form in rotifers, confirming the ability of rotifers to metabolize and chemically transform selenite into selemethionine. Several works in the literature suggest that levels of Se between 1.4 and 3 mg Se/kg DW in rotifers meet the requirements of fish larvae [127,132,133], and the copepod Se levels are reported to range from 3 to 5 mg Se/kg DW [113]. Therefore, it is recommended that rotifers be enriched with selenite, selemethionine, and selenized yeast at 2, 0.2, and 0.2 mg of Se per 10^6^ rotifers for 12, 1.5, and 2 h, respectively, in order to achieve copepod Se levels of 3 mg Se/kg DW and the high accumulation of selemethionine in rotifers [125]. However, Penglase et al. [134] and Ribeiro et al. [135] demonstrated that 2.1 and 3 mg of selenized yeast per 10^6^ rotifers, respectively, in 3 h enrichments were needed to achieve the copepod Se levels. In the case of *Artemia*, 3 mg of selenized yeast per million individuals and 12 mg/L in 3 and 4 h enrichments, respectively, were needed to achieve the copepod Se levels [15,133]. These contrasts were probably due to the presence of other ingredients such as lyophilized algae, fish oil, and other commercial enrichment formulations. Thus, it can be concluded that the estimation of the Se enrichment duration varies substantially according to the Se sources. Selenized yeast, an organic source of Se, can be produced by exposing yeast to sodium selenite [136], resulting in the accumulation of selenomethionine. Se from organic sources is regarded to be more bioavailable for fish than those from inorganic Se [125], and the enrichment of Se via selenized yeast resulted in high retention of Se in enriched rotifers for an extended period of time [134]. Therefore, the enrichment of rotifers with selenized yeast can be used as an excellent Se delivery method to confer health benefits to fish larvae.

Kim et al. [132] studied the effects of enriching microalgae *C. vulgaris* with Se (sodium selenite at 3.3 mg Se/kg DW) on sexual and asexual reproduction of rotifers. Improved population growth, fertilization rate, and resting egg formation were observed due to the antioxidant abilities of glutathione peroxidase (GPx). GPx, a selenoprotein, is a compound that maintains the mechanical stability of spermatozoa [137], thus influencing the rates of fertilization. The study presented a method to produce a high-density mass culture of rotifers. Another approach involves the use of Se in the form of selenium nanoparticles (SeNPs). SeNPs has similar efficiency as organic Se forms that exhibit lower toxicity [138,139] and possesses antibacterial properties [140]. It was reported that the enrichment of *Artemia* with 5 mg/L SeNP solution for 24 h was needed to achieve the optimal Se content (4 mg/kg) (dry matter) of the feed for fish larvae. The enrichment solution contained nano-elemental Se, produced on the basis of a new ascorbic acid reduction method. Furthermore, SeNP had been previously biosynthesized by various microorganisms including *Pseudomonas alcaliphila* [141], *Bacillus* sp. [142], *Zooglea ramigera* [143], and *Enterococcus faecalis* [140]. *Artemia salina* fed with SeNP-enriched *Yarrowia lipolytica* biomass showed improved growth, survival, and disease resistance against *Vibrio harveyi* [144]. The SeNP used in the study was synthesized from the incubation of *Y. lipolytica* cells with 4 mM sodium selenite for 48 h. However, it is necessary to note that the toxicity of SeNP is different from those of other Se species and is poorly understood. Indeed, a study suggested that exposure to SeNP caused malformations in Japanese medaka (*Oryzias latipes*) offspring [145]. It is known that Se supplementation through zooplankton enrichment can enhance fish growth [146], survival [121,127], and thyroid hormone status. Therefore, the use of Se from organic sources and SeNP of lower toxicity would be highly beneficial to fish larvae.

In conclusion, the concentration of Se in rotifers and *Artemia* is much lower than in copepods. Therefore, it is common to enrich live food with Se to meet the copepod Se levels. Enrichment of zooplankton with Se can be performed using different forms of Se such as selenite, selemethionine, selenized yeast, and SeNPs. It is recommended that rotifers be enriched with selenized yeast at 0.2 mg of Se per 10^6^ rotifers for 2 h to achieve the copepod Se levels. Meanwhile, it is recommended that *Artemia* be enriched with 3 mg of selenized yeast per million individuals for 3 h to achieve the copepod Se levels. Moreover, the enrichment of *Artemia* with 5 mg/L SeNP solutions for 24 h is recommended to achieve the optimal Se content. However, the presence of other ingredients in commercial enrichment formulations and enrichment duration may influence the final concentration of Se in zooplankton.

### 4.2. Iodine

Iodine (I) is a crucial component of thyroid hormone, responsible for fish metamorphosis [147]. Since marine fish larvae feed naturally on copepods, their nutrient levels are commonly used as a reference to indicate larval dietary requirements [6,42,113]. Copepod I levels are reported to range from 50 to 350 mg/kg DW, 10-fold higher on average than that of rotifers [113] and significantly higher than that of *Artemia*, which only range from 1.1 to 4.6 mg/kg DW [148]. The superior I content in copepods compared to *Artemia* suggests that assimilation of I by the consuming fish larvae could be the cause for a significant increase in the synthesis of thyroid hormone and in the whole body I level [148]. Enrichment with 400 mg/L sodium iodide for 1.5 h can increase the concentration of I in rotifers to 112 mg/kg DW, but the I concentration in fish larvae may not be affected by the I enrichment of the rotifers [127]. In contrast, Ribeiro et al. [18] reported 47.86 and 64.2 mg/kg wet weight (WW) I in rotifers and *Artemia*, respectively, after 3 h enrichment with sodium iodide (at either 100 or 200 mg/L, depending on the density of the zooplankton). Consequently, larval whole-body I content showed a significant increase when fed with the enriched zooplankton. In multiple studies, Lipiodol Ultra Fluid, an ethiodized oil, was used as the I source in the enrichment diet [114,147]. It is advisable that rotifers be enriched with Lipiodol from 52 to 392 mg/kg DW to acquire copepod I level [114]. With regard to *Artemia*, enrichment with 6.25 mg/kg WW Lipiodol for 24 h resulted in an I concentration of 318 mg/kg DW, which was within the range found in copepods [147]. Furthermore, the enrichment with Lipiodol resulted in high retention of I in enriched *Artemia* for up to 6 h, and the whole body I concentration in the consuming fish larvae was improved when fed on the enriched *Artemia*. A study demonstrated the enrichment of rotifers for 3 h with different I sources: thymol iodide, 3,5-diiodosalicylic acid, chelated iodine, kelp, and sodium iodide [149]. Chelated iodine was found to be a poor source of I for rotifer enrichment and failed to reach minimum copepod I levels. The level of I in fish larvae varies depending on the species of zooplankton, the form of I, and the enrichment method. Differences due to different rearing conditions between these studies should be considered. Moreover, whether fish larvae can absorb and regulate I in the form of iodide from seawater is still unknown [147]. Iodine from seawater may be sufficient in adult fishes but is probably still not enough to achieve adequate exogenous thyroxine (T4) tissue concentration [148]. It is known that the supplementation of I through zooplankton enrichment can improve fish thyroid hormone level [148] and survival [127], besides preventing goiter [18].

In conclusion, as the concentrations of I in *Artemia* and rotifers are reported to be much lower than the copepod I levels, it is recommended that *Artemia* and rotifers be further enriched with sodium iodide. It is recommended that *Artemia* and rotifers be enriched with 100 and 200 mg/L sodium iodide, respectively, for 3 h to meet the copepod I levels. Consequently, an improved larval whole-body I content was reported when *Artemia* and rotifers were fed with the enriched zooplankton. Furthermore, different sources of I such as Lipiodol, thymol iodide, 3,5-diiodosalicylic acid, and kelp can be good substitutes to sodium iodide for zooplankton enrichment. However, factors including the species of zooplankton, the form of I, the enrichment method, and the rearing conditions must be considered when preparing the enrichment emulsions.

### 4.3. Other Trace Metals

The level of other minerals in rotifers are lower than in copepods, with manganese (Mn), copper (Cu), and zinc (Zn) by two-, three-, and five-fold respectively on average [113]. In general, fish larvae fed with rotifers are likely to have limited Zn and Se concentrations, while Mn, Fe, Cu, cobalt (Co), and iron (Fe) generally meet the fish dietary requirements [150]. Shortage of Mn, Fe, and Co may occur in fish at the late larval stage when fed on non-enriched *Artemia* [150]. Thus, co-feeding *Artemia* with rotifers or enrichment is highly recommended in larviculture practices. Enrichment of rotifers with an organically bound mineral mix (I chelate, Mn proteinate, Cu proteinate, Zn proteinate, and selenized yeast) for 3 h can uplift rotifers to copepod levels of Mn, Cu, Zn, and Se by replacing 6% of the commercial rotifer enrichment diet [151]. Enrichment of rotifers with minerals bound to an ingestible particle was more effective than minerals in soluble forms [134]. Furthermore, the retentions of Mn, Cu, Zn, and Se in rotifers were high after 18 h storage in clear water [151]. High rotifer mineral retention is an important aspect of the commercial hatchery management to ensure the intended mineral quantity is consumed by fish larvae. Moreover, enrichment of microalgae with minerals has been proposed due to the inability of zooplankton to directly absorb and retain minerals from the culture media [20,93,152]. Feeding of rotifers with *Chlorella* is a more effective approach to deliver Zn and Cu due to the ability of *Chlorella* to absorb and pre-accumulate waterborne Zn and Cu [20,152]. The rotifer Zn and Cu contents were recorded at 373.2 and 50.5 mg/kg DW, respectively, when fed with enriched microalgae at 0.8 mg Zn/g and 0.1 mg Cu/g *Chlorella*, respectively, for 24 h [20,152]. The Zn content was within the range found in copepods (340 to 570 mg/kg DW), whereas the Cu content recorded in enriched rotifers was slightly higher than the copepod Cu levels (12 to 38 mg/kg DW) [113]. The constituents of *Chlorella* cell wall allow for the assemblage of ligands with different functional groups capable of binding different heavy metals [20]. The enhancement of the Fe content of *Artemia* through microalgae enrichment has been reported. Enrichment of microalgae *Tisochrysis lutea* with Fe (10 µg/mL) displayed a high content of Fe at 850 mg/kg DW, which was higher than the copepod Fe levels (85 to 371 mg/kg DW) [93]. It is important to note that the enrichment of zooplankton with one mineral may disturb the composition of the other minerals [20]. The literature suggest that zooplankton can be enriched with minerals by means of food ingestion rather than by immersion in enrichment media to promote better growth and physiological status of fish larvae in hatcheries. The positive effects of mineral enrichment to copepod levels in rotifers and *Artemia* were demonstrated in the larvae of red sea bream (*Pagrus major*) [153], Senegalese sole (*S. senegalensis*) [93], and Chinese Mitten crab (*Eriocheir sinensis*) [20,154].

In conclusion, fish larvae fed with non-enriched rotifers were likely to have limited Zn and Se concentrations. Fish at the late larval stage fed with non-enriched *Artemia* may experience a shortage of Mn, Fe, and Co. As the levels of other minerals including Mn, Cu, and Zn in rotifers and *Artemia* are much lower than in copepods, it is highly recommended that the zooplankton be enriched with minerals prior to larval feeding. Generally, the feeding of rotifers and *Artemia* with microalgae is regarded to be a more effective approach than the immersion method to deliver minerals to the zooplankton and fish larvae. Various studies on the applications of minerals in live food are compiled in Table 2.

## 5. Enrichment with Probiotics

The expansion of aquacultural activities together with environmental issues including climate change often contribute to conditions favoring disease outbreaks [155]. Disease is now a primary constraint to the culture of many aquatic species and therefore may put countries that rely heavily on fisheries for their livelihood in economic hardship or missed opportunities for development [156]. The conventional use of antibiotics for controlling bacterial infections is controversial and no longer effective in treating bacterial diseases in some cases [157,158,159,160]. Dietary administration of feed supplements such as probiotics to control or treat diseases has received increasing attention in recent years [161]. The term probiotics originated from the Greek words “pro bios” which mean “for life” [162]. In 1989, Fuller [163] revised the definition of probiotics to “a live microbial feed supplement which beneficially affects the host animal by improving its intestinal microbial balance”. Probiotics were then redefined as “live microorganisms which when administered in adequate amounts confer health benefits on the host” [164].

At the early development stage, artificial dominance of a specific cluster of bacteria can be stimulated in the host by the addition of a probiotic strain directly to the rearing water or the cultivation medium of the live food [165,166]. Administration of probiotics to the gut of the target host through probiotics enrichment in a bioencapsulation method of zooplankton live food is an interesting approach in some cases [167]. Bioencapsulation of live food with probiotics to enhance zooplankton growth, population density, and reproductive capacities has been reported [168,169,170,171,172,173,174].

Combined administration of two or more probiotic strains is considered to be more effective than single-strain administration in most cases [161]. *Artemia* accumulated the highest concentration of lactic acid bacteria (LAB) (5.22 × 10^3^ (colony-forming unit (CFU)/mL) when enriched with a mixture of three indigenous LAB (*Lactobacillus plantarum*, *Lactobacillus salivarus*, and *Lactobacillus rhamnosus*) at 10^7^ CFU/mL for 2 h [175]. Furthermore, the survival of crab larvae fed with either a single strain or a mixture of three LAB isolates via bioencapsulation was not significantly different between treatments but still higher than the control treatment. Thus, feeding of crab larvae with LAB-encapsulated zooplankton along with the direct addition of LAB to the rearing system may yield better results [176]. Generally, the administration of indigenous probiotic strains that include those from the normal dominant gastrointestinal microbiota of the host or any of its development phases is likely to yield dominant colonization [177]. Gatesoupe [178] reported the ability of three LAB strains to improve the production rate of rotifers. The mean concentration (150 rotifers/mL) and the production rate (34 rotifers/mL) were highest when enriched with 8 mg/L DW *L. plantarum* alone for 15 min every 6 h daily from day 6 until day 15. The spray drying method of whey culture medium with 10^6^ CFU/g *L. plantarum* was applied. The whey culture medium is often used as a carrier material for probiotic microencapsulation and generally contains lactose and soluble proteins [179]. However, a subsequent study found that enrichment of rotifers with two LAB strains between 10^7^ and 2 × 10^7^ CFU/mL once a day was enough to protect the consuming fish larvae from *Vibrio* sp. infection [180]. Despite the addition of probiotic mix (*Mycobacterium*, *Ruegeria*, *Pseudoalteromonas*, *Vibrio*) at 5 × 10^6^ CFU/mL directly to the rearing water together with the administration of rotifers and *Artemia* enriched with the equal mixture of the four probiotic strains at 4 × 10^8^ CFU/mL for 30 min, all strains were only transiently found in the larval microbiota [166]. The ability of the probiotic strains to grow in the planktonic state, or to establish biofilms in the tank walls and selective grazing by the zooplankton could affect the fate of the added strains, despite them being added in equal amounts [166]. The addition of single probiotic strain, however, is a far more common practice [181,182,183]. Enrichment of copepods with either lyophilized *Bacillus clausii* and *Bacillus pumilus* at 10^6^ CFU/mL for 3 h improved the growth performance, survival, and desirable gut microbiota of fish larvae [165]. The added probiotic strains are likely to aid in the modulation of intestinal digestive enzymes, lysozyme, and superoxide dismutase activities [165].

Nimrat et al. [184] assessed the effects of different probiotic forms and modes of probiotic administration on postlarval white shrimp (*L. vannamei*). *Bacillus* spp. was administered to shrimp culture in the form of freeze-dried, microencapsulated beads, and bioencapsulation of *Artemia*. *Artemia* was enriched with microencapsulated *Bacillus* spp. at 10^9^ CFU/mL for 6 h. Microencapsulated and freeze-dried *Bacillus* spp. significantly enhanced the growth and survival of post-larval shrimp. In another study, commercial *Bacillus* spp. was either (1) added directly to the rearing water, and/or (2) with probiotic-enriched *Artemia* at 2.2 × 10^7^ CFU/mL for 10 h [185]. Both studies suggested that the forms of the probiotic strains and the modes of probiotic administration did not influence the growth and survival of the post-larval shrimp. Moreover, it is important to note that the efficiency of probiotic strains is dependent on the duration of exposure [185]. A study assessed the effects of synbiotic enrichment of zooplankton [186]. Synbiotic enrichment (*Pediococcus acidlactici* at 700 mg/L and fructooligosaccharide at 100 mg/L) of *Artemia* significantly improved fish growth performance, microbial diversity, stress tolerance, and immune responses. Besides this, HUFA was also administered along with the probiotic-enriched zooplankton [187]. Administration of HUFA-rich emulsion (cod liver oil) at 0.5 mL/L together with commercial *L. sporogenes*-enriched *Artemia* at 10 mg/L for 6 h and then 12 h at day 10 improved the survival of prawn larvae. Interestingly, the larvae in the probiotic-enriched group contained DHA at low concentration (0.4%), while the larvae fed with *Artemia* enriched with both emulsion and probiotic had the highest contents (4.4%). It has been reported that *Shewanella putrefaciens*-enriched *Artemia* may contribute to the elevation of n-3 HUFA levels in the consuming fish larvae [188]. Added microbiota were proven to regulate the capacity of intestinal absorption and metabolism of fatty acids in fish [189]. The effects of combining a probiotic strain and a bacteriophage were assessed [190]. The addition of *P. inhibes* at 10^7^ CFU/mL and vibriophage at 10^7^ PFU/mL was able to inhibit the growth of *Vibrio anguillarum* and protect *Artemia* from vibrio infection. However, the interactions between the probiotic strain and vibriophage needed to be further studied as the vibriophage was unable to reduce the mortality of *Vibrio*-challenged *Artemia*, despite having the ability to initially lower *Vibrio* counts in *Vibrio* treatment alone. Thus, the quick reduction of vibrios in *Artemia* indicated that phage therapy followed by subsequent addition of probiotic could be a practical approach for controlling vibrios in zooplankton cultures [190].

Enrichment of live food with probiotics allows it to remain viable and proliferate in the live food constituents, and therefore it can be effectively transported into the hosts [191]. Probiotic bacteria are not only able to enhance the nutritional value of live food by providing essential compounds such as vitamins or inorganic nutrients lacking in the diet but are also able increase the population density of live food and inhibit the growth of pathogens [168,178]. Moreover, a direct administration of probiotics to the culture water is risky as they are easily exposed to microbiological contamination [165]. Furthermore, the short survival period of probiotics in seawater makes the utilization of live food as a vector an ideal approach [192]. Since live food stays in the rearing water for a few hours before it can be consumed, the bioencapsulated bacteria should be able to remain in the live food long enough prior to larval feeding [172]. Therefore, it is necessary to assess the rate of loss of the bioencapsulated bacteria and the tenacity of the altered bacterial composition [193]. At the early developmental stage of the fish larvae, the growth in the number of bacteria in the fish intestinal microflora is closely related to the bacteria in the live food [194]. Therefore, the enrichment of live food with probiotics in the bioencapsulation method allows for control of the bacterial population in the live food. This may lead to a better growth performance and survival of the fish and crustacean larvae [194].

In conclusion, the bioencapsulation of live foods such as *Artemia*, rotifers, and copepods with probiotics is a common approach to deliver probiotics to a wide range of fish and crustacean larvae. Even though the single probiotic strain administration is a far more common practice, several studies showed that the combined administration of two or more probiotic strains is more effective. It is very common to use *Bacillus* spp. and LAB such as *Lactobacillus* spp. in larviculture practice. Additionally, it is recommended that an indigenous probiotic strain be utilized, one that includes the normal dominant gastrointestinal microbiota of the host or any of its developmental phases. The application of the indigenous strain would be advantageous in terms of yielding more dominant colonization. Moreover, probiotic strains can also be administered along with prebiotics, HUFA, and bacteriophages to enhance the nutritional status of the zooplankton that consequently may confer health benefits to fish and crustacean larvae. The applications of probiotic-enriched live food in aquaculture are summarized in Table 3.

## 6. Conclusions

This review focused on the modification of the nutrient composition of zooplankton as live food through supplementation of essential nutrients in culture media before they are fed to fish and crustacean larvae. Live food acts as an important basic diet for larval cultivation, and the availability of appropriate quantities of essential nutrients in the larval diet is crucial to ensure the successful rearing of fish larvae. The enrichment of zooplankton live food through bioencapsulation is convenient to improve the nutritional status of live food for consumption by fish larvae. Encapsulation of nutrient-deficient live food such as rotifers and *Artemia* with micro- and macro-nutrients has been demonstrated to elevate the dietary value of the live food and to enhance the performance of fish larvae and fries. Rotifers and *Artemia* are commonly enriched with micro-nutrients to meet the copepod nutrient levels, which are frequently used as reference to indicate larval dietary requirements. The nutritional profile of copepods can be altered through changes in dietary algal nutrition by the pre-enrichment of algae with various essential nutrients. Even though live food is considered as “living capsules of nutrition”, further evaluations are needed to establish the stability and the high retention rate of nutrients in live food to ensure the intended quantity is successfully delivered to larval fish and crustaceans. On the basis of the literature we reviewed, we suggest that sustainable and effective larval rearing can be achieved, and with better understanding of the enrichment techniques, other sources of feed may gradually be used as substitutes to live food.

## Figures and Tables

**Table 1:** Enrichment of live food with vitamins.

Vitamin	Live Feed	Biological Model	Method	Effects	References
**Vitamin C**	*M. micrura*	Climbing perch (*Anabes testudineus*)	Co-enrichment with HUFA for 24 h	Growth promoter	[19]
	*Artemia* nauplii	Senegalese sole (*S. senegalensis*)	Co-enrichment with dietary Fe for 24, 29, and 33 h	Growth promoter	[93]
		Patagonian red octopus (*E. megalocyathus*)	Co-enrichment with algae for 2 h	Growth promoter and survival improvement (not significant)	[94]
		-	Enrichment with unilamellar liposomes composed of soybean phosphatidylcholine and loaded with sodium ascorbate	Low vitamin C content in the nauplii	[115]
	*Moina, Daphnia, Cyclops*, and *Diaptomus*	Rohu (*Labeo rohita*)	Enrichment with 20% ascorbyl palmitate for 12 h	Growth promoter and survival improvement	[117]
	*Artemia* nauplii and rotifer *Brachionus plicatilis*	Milkfish (*C. chanos*)	Co-enrichment with HUFA for 24 h	Growth promoter, survival improvement, and lower incidence of opercular deformity	[92]
	Rotifer *Brachionus* sp.	-	Co-enrichment with thiamine, vitamins A and E, and iodine	Enrichment with 4.6% Stay C in the diet could give copepod levels of vitamin C	[114]
**Vitamin A**	*Artemia* nauplii	Striped trumpeter (*L. lineata*)	Enrichment emulsions contain retinyl palmitate together with lipids, and vitamin E and C (24 h)	Growth and survival were not significantly affected by increasing dietary doses of retinyl palmitate	[118]
	*Artemia* metanauplii	Senegalese sole (*S. senegalensis*)	Addition of retinyl palmitate to a commercial enrichment emulsion	Survival improvement	[119]
	Rotifer *B. plicatilis*	Striped trumpeter (*L. lineata*)	Enrichment with retinyl palmitate for 2 h	Growth and survival were not significantly affected by increasing dietary doses of retinyl palmitate	[99]
				Retinyl palmitate enrichment in rotifers did not affect the type or severity of jaw malformations	
	Rotifer	Atlantic cod (*Gadus morhua*)	Co-enrichment with oil mixtures for 2 h along with the addition of fish meal, Selplex, and iodine	Alteration of the skeletal metabolism during larval development	[120]

**Table 2:** Enrichment of live food with minerals.

Mineral	Live Feed	Biological Model	Method	Effects	References
**Selenium**	*Artemia* nauplii	-	Enrichment with sodium selenite and selenoyeast at 24 µg/L for 4 h	Enrichment with selenoyeast increased the levels of Se in the nauplii	[15]
	*Artemia* sp.	Red drum (*Sciaenops ocellatus*)	Enrichment with nano-selenium	Promotion of growth and improved survival	[121]
	Rotifer *B. plicatilis*	-	Enrichment with selenite (2 mg), selenomethionine (0.2 mg), and selenized yeast (0.2 mg) for 12, 3, and 6 h, respectively	Rotifers with Se levels similar to those in copepods can be achieved	[125]
		Atlantic cod (*G. morhua*)	Co-enrichment with sodium selenite (7 mg/L) and sodium iodide (400 mg/L) for 1.5 h	Improved survival	[127]
		-	Enrichment with Se-fortified *C. vulgaris* (containing 3.3 µg Se/g DW) at 2.5 × 10^6^ cells/mL	Higher population growth, rates of fertilization, and absolute resting egg production of rotifers	[132]
		-	Enrichment with selenized yeast at 0.01, 0.02, 0.025, 0.04, and 0.08 g per million rotifers for 3 h	The Se levels obtained were higher than copepods Se levels	[135]
	*Artemia* nauplii and rotifer *B. plicatilis*	Senegalese sole (*S. senegalensis*)	Enrichment with 0.003 g of selenized yeast per million individuals and DHA Selco for 3 h	The activity of glutathione peroxidase and the production of thyroid hormone were higher	[133]
	Rotifer *Brachionus* spp.	-	Enrichment with selenized yeast at 1.7 mg for 6 days and 3.2 mg for 3 h	A high retention rate of Se for up to 10 h storage in clear water at cold (10 °C) or warm (20 °C) temperatures	[134]
	*A. salina*	-	SeNP enriched biomass of *Y. lipolytica at* 10^9^ cells/mL was added every 24 h	Promotion of growth, improvement in survival, and enhancement in disease resistance	[144]
	Rotifer *Brachionus rotundiformis*	Red seabream (*P. major*)	Enrichment with Se-fortified *C. vulgaris* (3.2 µg Se/g DW) at 5 × 10^5^ cells/mL	Promotion of growth and higher Se concentrations of rotifers and fish larvae	[146]
**Iodine**	Rotifer *B. plicatilis* and *Artemia*	Senegalese sole (*S. senegalensis*)	Enrichment with 780 mg sodium iodide per 1 g emulsions: Rich Advance (for rotifers) or Super Selco (for *Artemia*) for 3 h	Promotion of growth; moreover, the whole body I concentration of larvae fed the I-enriched live food was higher compared to control larvae	[18]
	Rotifer *Brachionus* “Cayman”	-	Rotifers were fed three meals daily (containing Lipiodol at either 100, 200, or 300 mg/kg DW feed) for 4 days	52 mg/kg dietary iodide would be needed to obtain the lower range of copepod levels of iodine	[114]
	Rotifer *B. plicatilis*	-	Enrichment with 200 mg sodium iodide per million rotifers and mixed with DC DHA Selco for 3 and 6 h	The I levels in the enriched rotifers were significantly higher than those of the control rotifers	[135]
	*Artemia* nauplii	Atlantic halibut (*H. hippoglossus*)	Enrichment with Lipiodol Ultra Fluid at 0.2 g/L for 24 h	Enhancement in the levels of I in fish larvae	[147]
	Rotifer *Brachionus* “Cayman”	-	Rotifers were enriched with a diet containing either thymol iodide, 3,5-diiodosalicylic acid (both at 9.7 g/kg rotifer DW), or sodium iodide (at 0.3 g/L) for 3 h	The levels of I in rotifers met copepods I levels	[149]
**Other trace metals**	Rotifer *Brachionus* sp.	-	Enrichment with yeast and oil, yeast and Algamac 2000, yeast and *Chlorella*, and Culture Selco	The I, Mn, Cu, Zn, Se, and Fe concentrations in rotifers were lower than the concentrations measured in copepods	[113]
	Rotifer *B. plicatilis*	-	Pre-accumulation of 1 mL zinc sulphate solution in 10 g *Chlorella* for 12 h. Followed by the incubation of rotifers with Zn-enriched *Chlorella* at 1.8 × 10^6^ cells/mL for 24 h	The Zn content of rotifers fed zinc-enriched *Chlorella* was significantly higher than that of rotifers fed unenriched *Chlorella*	[152]
	*Artemia* nauplii	Red seabream (*P. major*)	Prior enrichment of marine ω A with 0.1 mg Zn/mL or 0.24 mg Mn/mL for 2 h before incubated with *Artemia* for 32 h	Growth and normal skeletal development were promoted	[153]
		Chinese mitten crab (*E. sinensis*)	Prior enrichment of marine ω A with 0.1, 0.2, or 0.4 mg Cu/mL for 3 h before incubated with *Artemia* for 24 h	Promotion of growth, superoxide dismutase, and catalase activity enhancement, and improvement of salinity stress tolerance	[154]

**Table 3:** Enrichment of live food with probiotics.

Probiotic Strains	Live Feed	Biological Model	Method	Effects	References
*Bacillus* spp.	Copepod *Pseudodiaptomus annandalei*	Grouper (*E. coioides*)	Incubation with individual strain of lyophilized probiotic for 3 h	Growth promoter, survival improvement, and inhibition of pathogens	[165]
*Bacillus* spp.	*Artemia franciscana*	Pacific white shrimp (*L. vannamei*)	Incubation with mixed microencapsulated probiotics for 6 h	Growth promoter, survival, and water quality improvement	[184]
*Bacillus* spp., *Debaryomyces hansenii*, *Rhodotorula* sp., and *Chaetoceros* sp.	*A. franciscana*	Pacific white shrimp (*L. vannamei*)	Incubation with individual strain of microencapsulated probiotic for 6 h	Growth promoter and survival improvement	[195]
*Bacillus subtilis, Lactobacillus* sp., and *Lactococcus* sp.	*A. franciscana*	*-*	Single administration of each probiotic strain	Survival improvement	[196]
*B. subtilis, Lactobacillus* spp., and *Lactococcus* spp.	*Artemia* nauplii	*-*	Administration of mixed 10 probiotic strains	Inhibition of pathogens and survival improvement	[197]
*B. subtilis, Lactobacillus* spp., and *Pediococcus* spp.	Rotifer *B.* *rotundiformis* and *Proales similis*	*-*	Co-feeding a mixture of LAB and *B. subtilis* with algae paste	Growth promoter	[170]
*Bifidobacterium animalis, Lactobacillus johnsonii*, and *Bacillus* sp.	*Artemia* metanauplii	Shortfin silverside (*Chirostoma humboldtianum*)	Incubation with individual probiotic strain for 40 min	Growth promoter and survival improvement	[198]
Commercial probiotic products	Rotifer *B. rotundiformis*	*-*	Co-feeding with *N. oculata*	Growth promoter	[199]
Commercial probiotic products	Rotifer *Brachionus calyciflorus*	*-*	Co-feeding with *C. vulgaris*	Growth promoter	[200]
Commercial probiotic products and pure isolates	Rotifer *B. plicatilis*	*-*	Co-feeding either with artificial diet or axenic microalgae or both	Growth promoter	[168]
*Escherichia coli*	*Artemia* nauplii	Black tiger shrimp (*Penaeus monodon*)	Enrichment with *E. coli* expressing dsRNA-LSNV for 2 h	Elimination of viral infection	[201]
LAB strains	Rotifer *B. plicutilis*	-	Individual or joint addition of several strains	Growth promoter	[171]
*Lactobacillus sporogenes*	*Artemia* nauplii	Freshwater prawn (*Macrobrachium rosenbergii*)	Incubation with the lyophilized probiotic strain for 12 h	Growth promoter and survival improvement	[182]
			Incubation with the probiotic strain suspension (sporolac tablet) for 7 h	Growth promoter	[202]
*Lactobacillus* spp.	Rotifer *B. plicatilis* and *A. franciscana*	Blue swimming crab (*Portunus pelagicus*)	Incubation with a single or multiple probiotic strain for 2 h	Inhibition of pathogens and survival improvement	[175]
*Mycobacterium*, *Ruegeria*, *Pseudoalteromonas*, and *Vibrio*	Rotifer *Brachionus ibericus* and *Artemia* nauplii	Atlantic cod (*G. morhua*)	Incubation with equally mixed probiotics for 30 min	Probiotic strains are only transiently present in larva	[166]
*Phaeobacter inhibens*	*A. salina*	-	Co-culture of *Artemia*, non-axenic algae, the probiotic strain, and pathogen for 96 h	Inhibition of pathogens and survival improvement	[190]
*Phaeobacter* sp.	Rotifer *B. plicatilis*	-	Enrichment with algae and the probiotic strain for 24 h	High probiont retention for 48 h after enrichment	[172]
*Saccharomyces boulardii*	*Artemia* nauplii	-	Incubation at 3 different concentrations for 24 h	Survival improvement	[181]
*Strain* 4:44 and PB52	Rotifer *B. plicatilis*	Turbot (*Scophthalmus maximus*)	Incubation with either a single or mixed strain for 20 min	Successful colonization of the gut	[193]
*Weissiella koreensis*	*Artemia* nauplii	Stellate sturgeon (*Acipenser stellatus*)	Incubation for 10 h	Growth promoter and survival improvement	[203]
